# Culinary continuity in central Japan across the transition to agriculture

**DOI:** 10.1007/s12520-024-01992-9

**Published:** 2024-06-07

**Authors:** Jasmine Lundy, Manon Bondetti, Alexandre Lucquin, Helen M. Talbot, Natsuki Murakami, Seiji Nakayama, Motoki Harada, Miho Suzuki, Eiko Endo, Chris Stevens, Enrico R. Crema, Oliver E. Craig, Shinya Shoda

**Affiliations:** 1 Department of Archaeology, BioArCh, University of York, York, YO10 5ND UK; 2 Nara National Research Institute for Cultural Properties, Nara, 630-8577 Japan; 3 Research Institute of Cultural Properties, Teikyo University, Yamanashi, 406-0032 Japan; 4 Aichi Asahi Site Museum, Kiyosu, Aichi 452-0932 Japan; 5 Centre for Obsidian and Lithic Studies, Meiji University, Tokyo, 101-8301 Japan; 6 McDonald Institute for Archaeological Research, University of Cambridge, Cambridge, CB2 3ER UK; 7 Department of Archaeology, University of Cambridge, Cambridge, CB2 3DZ UK

**Keywords:** Organic residue analysis, Jōmon pottery, Yayoi pottery, Agriculture, Central Japan

## Abstract

Rice and millet arrived in Western Japan from Korea around 3,000 years ago and spread eastwards across the archipelago in the next 700 years. However, the extent to which agriculture transformed traditional Jōmon hunter-gatherer-fisher communities is debated. Central Japan is a key area of study as remodelling of radiocarbon dates shows a slowdown in the dispersal rate of rice agriculture in this area. Here, we examine and compare the use of pottery by Final Jōmon and Early to Middle Yayoi communities in the Tōkai and the Central Highland regions of central Japan, using lipid residue analysis. Although the identification of specific biomarkers for rice remains elusive, an increase in the ratio of E/H C_18_ APAAs with the arrival of rice and millet indicates a potential change in plant processing and consumption. We were also able to identify biomarkers for broomcorn millet (miliacin) in both Final Jōmon and Yayoi pottery. However, evidence for millet consumption is sparse and in all cases was likely mixed with wild hunted and foraged foods. We conclude therefore that, despite the introduction of rice and millet agriculture in central Japan, pre-existing diets and culinary habits of Jōmon hunter-gatherers remain important.

**Supplementary Information:**

The online version contains supplementary material available at 10.1007/s12520-024-01992-9.

## Introduction

Japan has one of the richest, most well-studied ceramic sequences in the world that dates back to the late Pleistocene (Aikens 1995; Taniguchi 2017). In the absence of widespread organic preservation, particularly impacting the preservation of human and animal bone due to prevailing acidic soils, ceramics have been fundamental to understanding key cultural changes across the Japanese archipelago. In the last decade, it has been shown that lipids (fats, waxes, and oils) can be extracted from even the very earliest pots to reveal information regarding their use, providing insights into the economy, environment, and culinary practices of the prehistoric hunter-gatherers of Japan (Craig et al. 2013; Lucquin et al. 2016b, 2018). The majority of this research has so far focused on hunter-gatherer pottery of the Jōmon period (ca. 16.0k − 2.9k BP), while the transition to a farming economy that marks the end of this cultural sequence has received less attention. The arrival of rice cultivation along with a series of interrelated innovations, including paddy fields, new toolkits, and changes in settlement patterns, traditionally marks the end of the Jōmon period and the beginning of the Yayoi period (Sahara 1975; Hashiguchi 1987; Mizoguchi 2013). Although there is a substantial focus on rice, both foxtail (*Setaria italica*) and broomcorn (*Panicum miliaceum*) millets were also important components of the Yayoi cultural package (Shitara 2023). Importantly, the social, economic, and cultural transformation of the Yayoi transition is thought to be geographically diverse. From a chronological standpoint, this also means that the transition from the Jōmon to Yayoi periods occurred at various times in different regions, with the earliest transition occurring at the turn of the 1st millennium BCE in Northern Kyushu and the latest in Northern Kanto about 700 years later (Crema et al. 2022).

The typo-chronological pottery sequence that defines the Final Jōmon and Early Yayoi is complex, and investigations on its absolute dating have been limited by the fact that the main dispersal of the Yayoi package and the consequent local transitions from Jōmon to Yayoi occurred during the 800–400 BCE radiocarbon calibration plateau. In central Japan, rice and millet impressions are occasionally found on Final Jōmon/ Initial Yayoi pottery types, e.g. *Fusenmon I* and *Tottaimon* (Endo and Leipe 2021) that pre-date the appearance of typical Early Yayoi ‘*Ongagawa’* style cooking pots, traditionally associated with agricultural expansion (The Japanese Archaeologists Association 1981). Similarly, vessels that retain Jōmon features, such as *Jokonmon* style vessels, continued to be produced alongside *Ongagawa* vessels during the Early Yayoi period (c.500 BCE to c 350 BCE in Central Japan). While understanding the relationship between pottery styles and the arrival of agriculture is important, so far, only limited studies have considered the actual uses of pottery across the sequence, mainly based on their form and volume (Sato 1999, 2015; Kobayashi 2004) or use wear analysis (Kobayashi 2003; Tokusawa et al. 2007). Additionally, bulk isotope analysis of food crusts adhered to Yayoi pottery has been used to evaluate the extent of C_4_ plant consumption, but this method alone provides only a crude resolution of vessel contents (Sakamoto 2007). The existence of different ceramic typologies at individual sites offers the opportunity to directly distinguish their use and to test whether typology follows function.

Here, for the first time, we compare the use of Final Jōmon and Early to Middle Yayoi pottery (12th C to 1st C BCE) from the Central Highlands and the Tōkai regions of Japan using lipid residue analysis to examine the impact of the arrival of the Yayoi ‘package’ on local culinary tradition. A fundamental question is to determine whether Early Yayoi pottery was used for cooking rice and millet, implying that pottery styles and food production were closely linked, or whether there was a continuation in culinary practices from the Jōmon period, with vessels used for cooking wild plant resources, fish, and game (Craig et al. 2013; Lucquin et al. 2016b). Addressing this question has wider implications regarding the economic and cultural impact of the Jōmon/Yayoi transition and in determining whether this was primarily driven by the adoption and expansion of the new economy by migrant groups from Korea or by the expansion of agricultural groups already established in Western Japan at this time (Barnes 2019; Hudson et al. 2020; Stevens et al. 2022).

### Understanding the arrival of rice and millet in central Japan

Rice and millet first appeared in northern Kyushu at the turn of the 1st millennium BCE (Miyamoto 2018; Obata and Kunikita 2022) and broadly spread through the archipelago from West to East Japan, but with regionally diverse rates of dispersal (Crema et al. 2022). The Chubu region, central Japan, marks the western margin of the Final Jōmon *Ōbora* and *Fusenmon I* pottery traditions distributed throughout eastern Japan and the eastern margin of *Tottaimon* pottery distributed throughout western Japan (Misaka and Wakabayashi 2011). A recalculation of the recent Bayesian analyses of radiocarbon dates shows that there was a delay of around 200–300 years in the dispersal of rice from Kansai (western Japan) to Chubu (central Japan), and a further 400–500 years delay from Chubu to Kanto (eastern Japan) (Crema et al. 2022; fig. S21), highlighting the importance of this transitional area that divides the East and West.

**Fig. 1:**
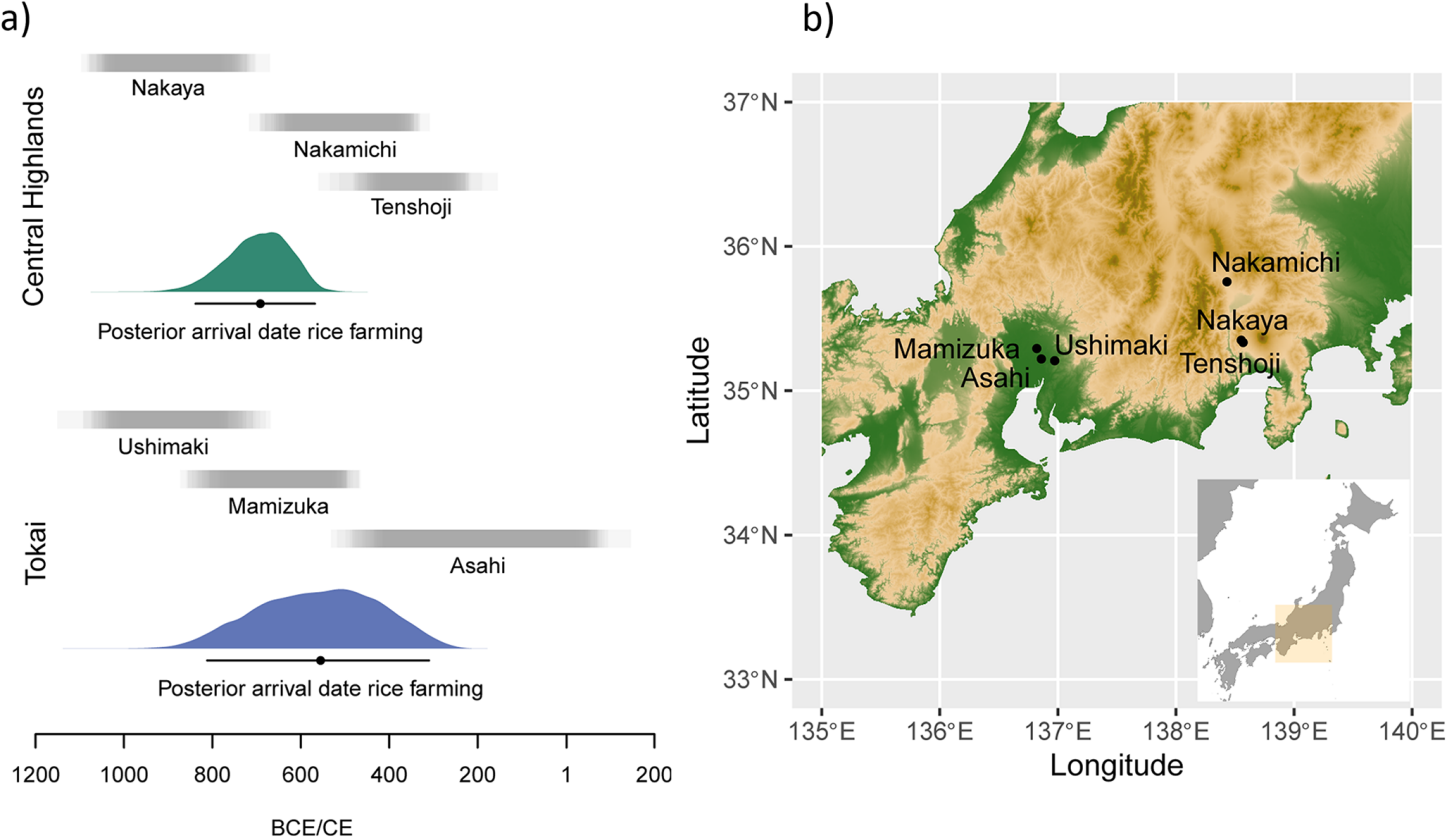
(**a**) Estimated date ranges of each site based on (Nishimoto 2007; p. 185) with the estimated arrival times of rice farming; (**b**) sampling site locations

The Chubu region is made up of contrasting topographic landscapes characterised by coastal plains in the western Tōkai region and mountainous settings in the eastern ‘Central Highlands’. To further understand the arrival of rice and millet in central Japan, here we have remodelled radiocarbon dates and estimated the arrival time of rice in Tōkai and the Central Highlands. Results indicate a substantial overlap, in part, due to the reduced sample sizes, with Tōkai dated between 811 and 310 BCE (95% HPD) and the Central Highlands between 838 and 568 BCE (95% HPD) (Fig. 1a) (Online Resource 1). Additionally, studies on pottery impressions in this region have questioned the synchronous arrival of rice and millet; the evidence at hand suggests that the arrival of millet in the region precedes that of rice, and has been explained by the topographical suitability for dry crops in the Central Highlands (Endo and Takase 2011; Fujio 2021). Our re-analyses of the data published by Endo and Leipe (Table 1), show that in the Central Highlands, both millet and rice impressions increase over time, with higher frequencies of millet across all periods. Evidence from Tōkai is more limited due to the smaller sample sizes, but by the Middle Yayoi period, our data indicates that rice impressions were higher than millet (Online Resource 1). While the frequency of seed impressions is not a reliable proxy for the absolute intensity of crop consumption, and it should be noted that these results only consider ceramics from the Late Jōmon onwards, these results highlight the generally low number of impressions per potsherd but with some distinct trajectories in the two regions.

**Table 1:** Posterior median (in bold) and 95% highest posterior density interval (in brackets) of the estimated number of rice and millet seed impressions per potsherd in the two regions across the main cultural phases examined. Estimates were obtained via a hierarchical negative binomial model (see Online Resource 1 for additional details)

		Rice	Millets	N.sites	N.sherds
Central Highlands	Late Jōmon - Initial Yayoi	**0.0008** (< 0.0001–0.0178)	**0.0156** (< 0.0001–0.8626)	3	4,468
	Early Yayoi	**0.0018** (0.0001–0.011)	**0.1468** (0.037–0.4321)	15	22,196
	Middle Yayoi	**0.0279** (0.0116–0.0582)	**0.4099** (0.1186–1.0922)	19	7,465
Tōkai	Late Jōmon - Initial Yayoi	**< 0.0001** (< 0.0001 – < 0.0001)	**< 0.0001** (< 0.0001 – <0.0001)	1	523
	Early Yayoi	**0.0133** (0.0005–0.2716)	**0.2246** (0.0023–33.0737)	2	767
	Middle Yayoi	**0.0524** (0.0016–1.2797)	**< 0.0001** (< 0.0001 – < 0.0001)	2	878
	TOTAL	**–**	**–**	42	36,297

In terms of the impact of the arrival of agriculture, stable isotope analysis of human bone collagen can provide insights into the degree of consumption of C_4_ plants (such as millet), as these are relatively enriched in ^13^C compared to other terrestrial dietary components. In the Central Highlands, there is evidence that C_4_ plants were at least a minor dietary component from the later phases of the Final Jōmon period (Yoneda et al. 2021), no Yayoi human remains are available for comparison from this region. In Tōkai, numerous individuals have been analysed from coastal shell middens (Kiriyama and Kusaka 2017; Kusaka et al. 2018), some of which are contemporary with early Yayoi settlement in this region, but these sites lack *Ongagawa* pottery. Determining the degree of C_4_ plant consumption by individuals with a prevailing marine diet is confounded by similar δ^13^C endpoints. Additionally, both rice and millet are less likely to be reflected in collagen stable isotope values due to their relatively lower protein content compared to marine and other terrestrial animal products. Thus, an alternative approach is required.

### Detecting the processing of rice and millet through organic residue analysis

Organic residue analysis (ORA) offers a direct approach for the identification and utilisation of organic products. The ability to identify products that have been processed in pottery not only enables further evidence for their presence in Japan, but offers a unique perspective into how these resources were utilised and processed by populations (Craig et al. 2013; Horiuchi et al. 2015; Lucquin et al. 2016b, 2018). By understanding their culinary importance, it is possible to glean insight into the impact of the introduction of resources to everyday life.

To assess the impact of agriculture through ORA it is important to consider our ability to identify rice and millet in archaeological ceramics. Identifying plant products in archaeological ceramics, particularly starchy cereals and grains, is often hindered by their low lipid yield and non-specificity of biomarkers (Reber and Evershed 2004; Hammann and Cramp 2018; Shoda et al. 2018). However, recent advances in ORA methods have enabled the identification of a variety of plant products in archaeological ceramics. Broomcorn millet (*Panicum miliaceum*) has been readily identified in archaeological ceramics through the presence of miliacin (olean-18-en-3β-ol methyl ether), a pentacyclic triterpene methyl ester dominant in broomcorn millet grains (Bossard et al. 2013; Heron et al. 2016). Whilst specific to broomcorn millet, it is widely agreed that both foxtail millet and broomcorn arrived in Japan together at the same time (Nasu and Momohara 2016), and, although we cannot be certain, it is assumed that they are likely to have had similar culinary roles. Thus, miliacin provides a robust biomarker to identify the presence and processing of millet in archaeological ceramics if preservation allows.

Conversely, the detection of rice in organic residues is far more elusive. The identification of γ-tocopherols, sitosterols, and pyrolysis products of starch or cellulose (levoglucosan (1,6-anhydro-b-D-glucopyranose) have been proposed to indicate the processing of starchy plants in East Asian pottery, but these are not specific to rice (Shoda et al. 2018). Furthermore, the survivability of these compounds in other contexts is not well understood. Nonetheless, here we utilise a multifaceted organic residue analysis approach, by combining targeted extraction and analytical methods to aid our identification, not only of plants, but resources such as aquatic and terrestrial animal products also (see methods). For the first time, in combination with other lines of evidence, as outlined above, (seed impression data, macrofossil data, and human isotope data), we aim to understand when and how rice and millet were processed at the arrival of agriculture in central Japan and how it impacted culinary habits, by understanding whether there was a change in the use of pottery after the introduction of agriculture.

## Materials and methods

### Archaeological sites and samples

A total of 254 ceramic sherds were sampled for lipid residue analysis from 6 sites across the Chubu region, central Japan, from the contrasting geographical landscapes (Tōkai and the Central Highlands) (Fig. 1a, b). In the Tōkai region, two sites (Mamizuka and Asahi) on the alluvial land (Nobi Plain) and one site (Ushimaki) on the plateau were selected (Board of Education of Aichi Prefecture 1982; Aichi Prefecture Archaeological Research Centre 2001; Ichinomiya City Board of Education 2014; Aichi Prefectural Board of Education 2021). Three further inland sites were selected from the Central Highlands (Nakaya, Nakamichi and Tenshoji sites) (Nirasaki City Board of Education 1986; Yamanashi Prefecture Archaeological Research Centre 1996; Yamanashi Prefecture 2007). The sites from both Tōkai and the Central Highlands were selected based on their chronological relation to the estimated arrival time of rice agriculture (i.e., before, during, and after the transition) (Fig. 1a).

From Tōkai, Ushimaki and Mamizuka both provided late Final Jōmon vessels for analysis (*n =* 35 and 35, respectively). Ushimaki clearly dates prior to the arrival of agriculture in Tōkai, while Mamizuka can be placed slightly later, within the 95% HPD interval of the estimated arrival time of rice (Fig. 1a). Early Yayoi pottery is also present at Mamizuka but was unavailable for analysis here. Ceramic samples (*n* = 108) were also obtained from Asahi, an extensive settlement site on the Nobi plain spanning from the Early to the Final Yayoi period. Pottery samples of various forms (cooking pots, bowls, and jars) of both *Ongagawa* and *Jokonmon* styles were obtained from the Early and Middle Yayoi contexts of this site.

From the Central Highlands, ceramic samples were obtained from Nakaya, Nakamichi, and Tenshoji sites. Nakaya pre-dates the estimated arrival of agriculture in the highlands, and the analysed samples consisted of cooking vessels dated to the early Late Jōmon period (*n* = 30). Samples from Nakamichi site, which has an occupational span overlapping the estimated arrival time of rice in the region, were obtained from the late Final Jōmon period (*n* = 15). Finally, Early to Middle Yayoi ceramic samples (*n* = 15) were obtained from the site of Tenshoji, located in very close proximity to Nakaya and dated to after the arrival of rice agriculture in the region. Further details of samples are given in Online Resource 2.

### Organic residue analysis of archaeological samples

All samples were subject to an acidified methanol extraction (AE), as described in full elsewhere (Online Resource 1)(Courel et al. 2020; Dolbunova et al. 2023). Total lipid extraction (TLE) was conducted on a selection of samples (50%) using DCM: MeOH (2:1 V/V) mixture to investigate the presence of any intact wax esters or mono, di, triacylglycerols and sterols, tocopherols and starch products (Evershed et al. 1992; Shoda et al. 2018). Both sets of extracts underwent analysis by Gas Chromatography-Mass Spectrometry (GC-MS) where selected ion monitoring (SIM) protocols helped to further identify specific biomarkers for heating transformation markers (APAAs), aquatic biomarkers (isoprenoids) (Evershed et al. 2008; Cramp and Evershed 2014), markers for cereals (alkylresorcinols) (Colonese et al. 2017), and miliacin as a biomarker for millet (Heron et al. 2016). Gas Chromatography-combustion-Isotope Ratio Mass Spectrometry (GC-c-IRMS) was also applied to the AEs to further identify the origin of these lipids by measuring the δ^13^C isotope values of the dominant fatty acids (FAs) in the sample (FAC_16:0_ and FAC_18:0_). GC-c-IRMS can help to distinguish freshwater and marine values against terrestrial values as well as between C_4_ and C_3_ plant products based on the known isotopic values of archaeological and modern specimens from Japan as previously reported (Lucquin et al. 2018). Additional values were obtained from modern reference samples here, as described below. Full details of the method are outlined in Online Resource 1 and modern authentic reference values are provided in Online Resource 3.

### Modern reference samples

Thirteen samples of modern, authentic rice and millet grains were acquired from different provenances in Japan. These were heated in the laboratory in the presence of ceramic powder, as described elsewhere (Bondetti et al., 2021a), to form *ω*-(*o*-alkylphenyl)alkanoic acids (APAAs). The ceramic powder and heated plant samples were then extracted using the same AE method as described above. These extracts were analysed by GC-MS with selected ion monitoring mode (SIM) to measure the distribution of isomers of APAA-C_18_ and compared with previously published data (Bondetti et al. 2021b). The δ^13^C isotope values of C_16:0_ and C_18:0_ was also measured using GC-c-IRMS, corrected for the Suess effect, and compared to the results from the archaeological samples (Online Resource 1 and Online Resource 3).

## Results and discussion

### Molecular evidence for rice and millet

**Fig. 2:**
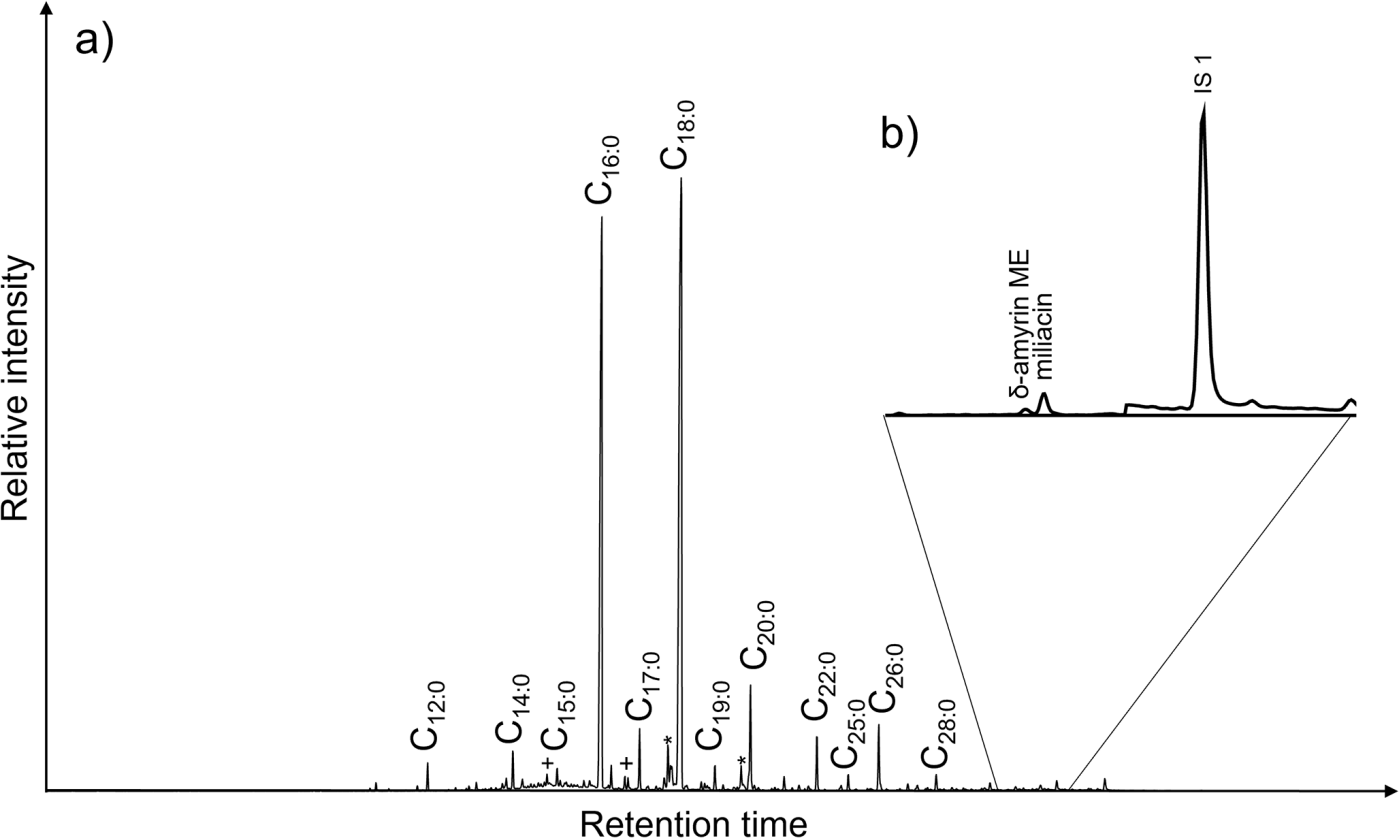
(**a**) Total ion chromatogram of AE of sample TSJ_001 showing the main saturated fatty acids (SFAs) identified whereby * indicates the unsaturated FA of the associated SFA and + indicates the branched isomers of the associated SFA. (**b**) Partial chromatogram of miliacin SIM showing amyrin methyl ester (ME) and miliacin (Online Resource 1). IS1 indicates the C_34_ alkane internal standard

Miliacin was identified in a total of 11 out of 254 samples (Asahi *n* = 2, Nakamichi *n* = 2, Tenshoji *n* = 7), using scan and/or SIM MS protocols (Fig. 2), providing direct evidence of the incorporation of millet into culinary habits in both the Tōkai and Central Highland regions. These findings are in broad agreement with other lines of evidence, such as seed impressions on pottery (Endo and Leipe 2021), and stable isotope evidence for millet consumption at other Final Jōmon sites in this region (Yoneda et al. 2021). Miliacin was always accompanied by lipids derived from other sources, notably animal fats, indicating either episodic mixing of millet with other ingredients or that the vessels had multiple inputs over their use-life. There is no evidence that millet was the main constituent or used exclusively in particular vessels, as has been occasionally observed in other studies (Ganzarolli et al. 2018).

Evidence for rice processing was limited across the study. None of the 129 TLEs analysed contained γ-tocopherols, a major component abundant in rice bran, or the pyrolysis products of starch, levoglucosan. These compounds were observed previously in pottery and food crusts from the waterlogged Tianluoshan site in eastern China, associated with early rice agriculture (Shoda et al. 2018). Their absence here might be attributable to the different depositional environment that is not conducive to their preservation.

**Fig. 3:**
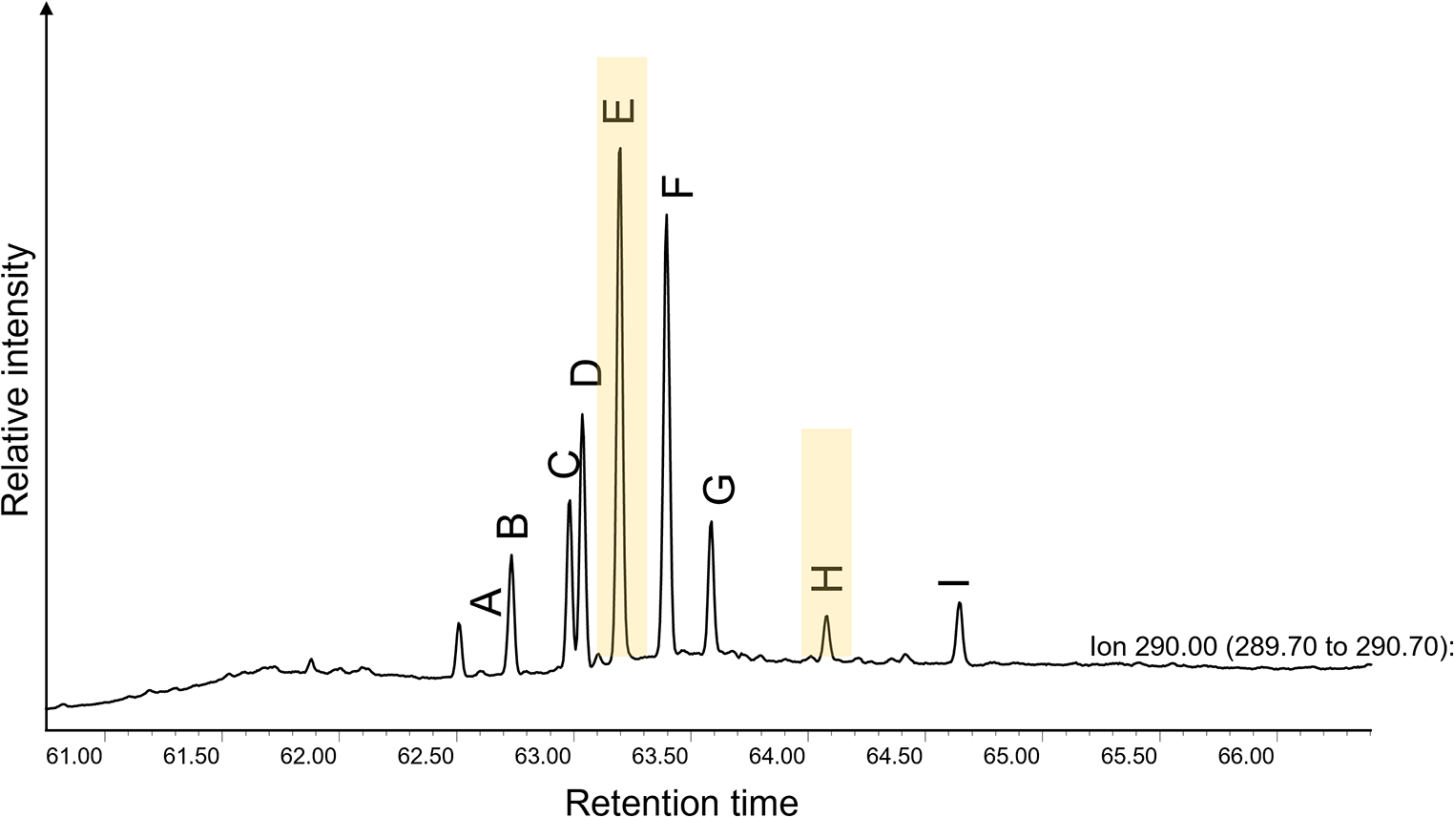
Partial ion chromatogram of sample ASA_011 showing the positional isomers (**A**–**I**) of APAA-C_18_ acquired using selected ion monitoring (SIM) for *m/z* ions 290. Positional isomers E and H used to calculate the isomeric E/H ratio are highlighted

**Fig. 4:**
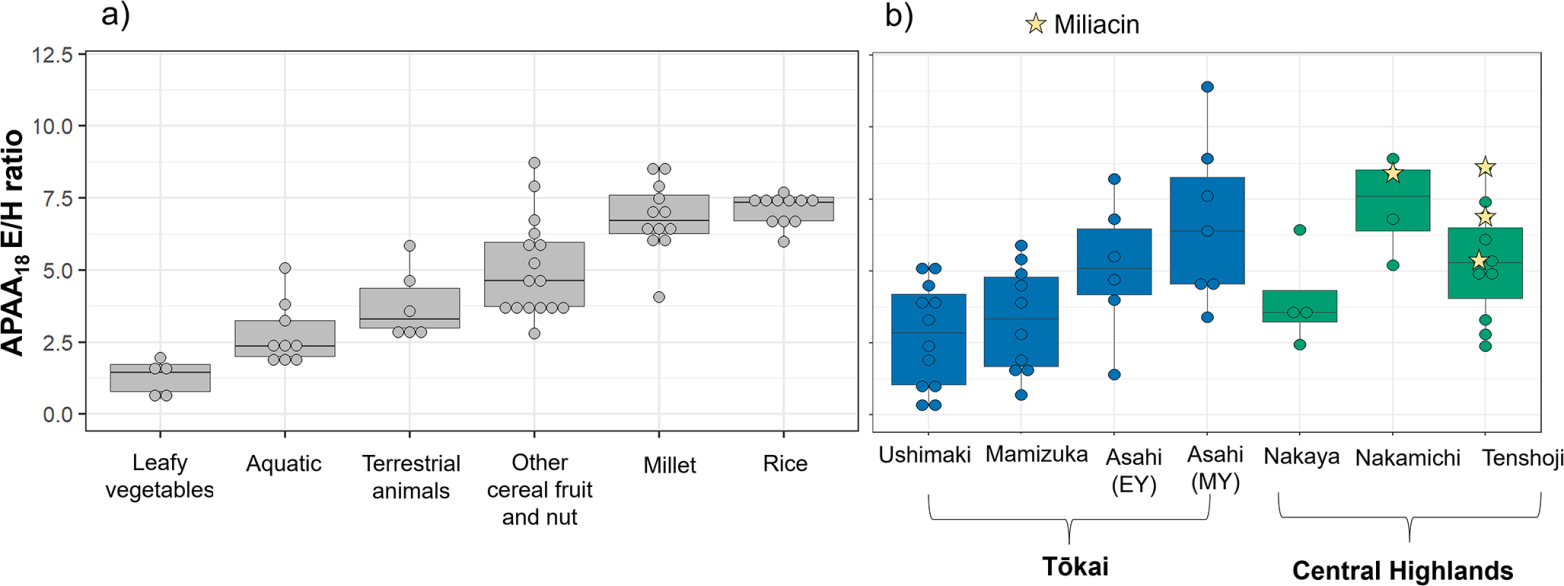
Box and dot plot showing the isomeric E/H ratio of APAA-C_18_ of (**a**) authentic modern products (Online resource 3 and (Bondetti et al. 2021b) and (**b**) archaeological samples from the 6 sites analysed in this study. Stars indicate samples with APAAs that yielded miliacin

One class of compounds, ω-(o-alkylphenyl)alkanoic acids (APAAs), was frequently encountered across the different pottery assemblages (*n* = 51). These are formed through the thermal transformation of mono- and polyunsaturated fatty acids that are present in animal fats, plant products and aquatic oils. Of these, the C_18_ homolog was most frequently identified. By assessing the distribution of the positional isomers (A-I, Fig. 3) of APAA-C_18_, it is possible to gain further insight into the origin of these heated products, particularly the ratio of the E and H isomers (Bondetti et al. 2021b). Here, we complemented the previously published dataset with experimentally heated rice and millet samples obtained from organic farms across Japan and the Himalayas. Analysis of these consistently produced relatively high APAA-C_18_ isomeric E/H ratios (Online Resource 3. i.e., > 6) compared to other plant and animal products, providing a crude means for their identification (Fig. 4a).

Next, we compared the isomeric E/H ratio of APAA-C_18_ of 51 archaeological samples with this expanded reference dataset (Fig. 4). Samples from Early Yayoi and Middle Yayoi phases at Asahi, Late Jōmon Nakamichi and Early Yayoi Tenshoji overall produced the highest APAA-C_18_ E/H values. Whereby, a number of samples (7 from Asahi, 1 from Nakaya, 3 from Nakamichi and 3 from Tenshoji) yielded values above 6. No statistical difference could be observed in values from the Central Highland sites. However, isomeric E/H ratios were statistically different between sites in Tōkai (Kruskal-Wallis chi-squared = 9.9753, df = 2, p-value = 0.006822). A post-hoc Dunn’s test revealed that the values obtained from Asahi are statistically distinguishable from those at Ushimaki, and Mamizuka (10.5281/zenodo.11164557). Samples that yielded miliacin and had preserved APAA-C_18_ yielded E/H values above 6 providing correspondence. Samples without miliacin but with high APAA-C_18_ E/H ratios might indicate the processing of rice or other starchy plants instead, but similar values produced by modern millet means it is difficult to distinguish the origin of APAA’s between the two grains here (rice and millets).

### Molecular evidence for other products

Evidence of processing fish and/or other aquatic organisms was also observed. Eighteen samples yielded APAAs with 20 and/or 22 carbon atoms meeting the criteria for heated aquatic oils (Bondetti et al. 2021a). These included samples from all sites except Nakamichi. We found no clear evidence for a higher consumption of aquatic resources in sites located on the Tōkai coastal plain (Online Resource 2). C_20_ and C_22_-APAAs are formed through heating aquatic oils and are less abundant than C_18_ homologs. It is therefore possible that this is an underestimation of the aquatic input. Isoprenoid fatty acids (phytanic, pristanic acid and TMTD (4,8,12-trimethyltridecanoic acid) occurring in aquatic oils were also frequently observed at each site. Of these, the ratio of the two diastereomers of phytanic acid (SSR%) was above 75% in a further 35 samples, suggesting the presence of aquatic products (*n* = 53) (Cramp and Evershed 2014; Lucquin et al. 2016a) (Online Resource 2). Notably, two samples from Tenshoji yielded biomarkers for broomcorn millet (miliacin) and aquatic resources (APAA _18/20_ > 0.06), providing evidence that these ingredients were mixed in a single recipe or different products were sequentially processed in these pottery sherds.

Further molecular evidence of plant products, such as Pinaceae products was identified in several samples across all 6 sites (Online Resource 2). Acetic acid and oxidation by-products (mainly dehydroabietic acid, 7-oxo-dehydroabietic and 15-hydroxy-dehydroabietic acid) were identified in a number of samples indicating either the intentional use of pine products as a sealant or, considering there is little other evidence for the use of conifer resins in pottery from Japan, exposure of the pot to conifer firewood during firing or cooking (Reber et al. 2019). Other terpenes such as lupeol derivatives were also identified in at least one sample (MMZ_023), possibly derived from other types of fuel or birch products. Additionally, long-chain fatty acids were also identified in several samples and, in most cases, a distinctive long-chain fatty acid profile with a predominant C_24_ was observed which likely derives from plant products (Dunne et al. 2022). Furthermore, in several cases, C_18:1_ was dominant, particularly in samples from Ushimaki and Mamizuka (Online Resource 2). Although a plant origin is highly likely, it is difficult to infer the specific origin of these long-chain and unsaturated fatty acids, and interpretations based on relative proportions of fatty acids in archaeological contexts should always be treated with caution.

### Comparison of pottery use between Tōkai and the Central Highlands

**Fig. 5:**
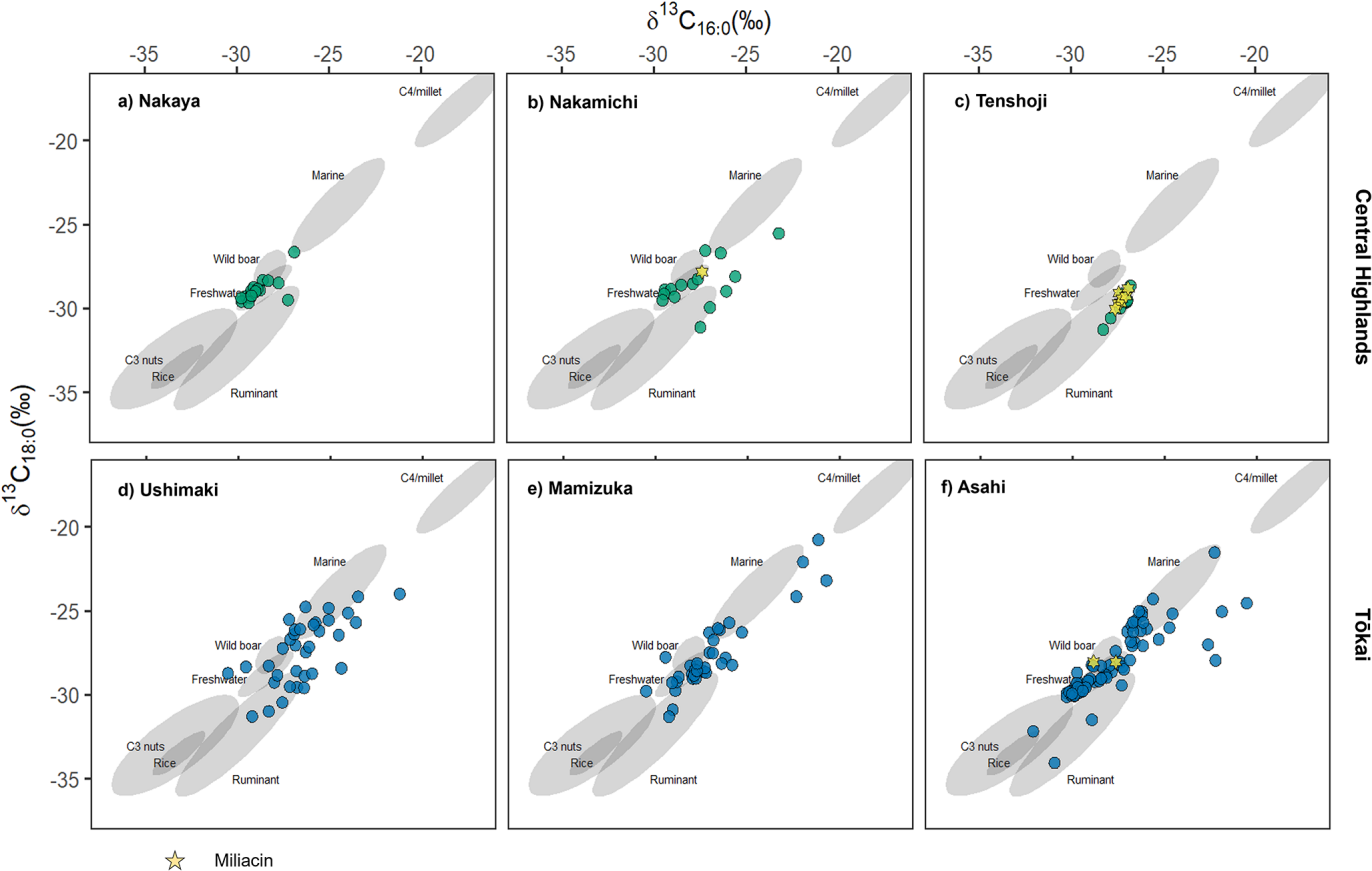
Plot of δ^13^C_16:0_ against δ^13^C_18:0_ of pottery extracts from Central Highland sites (**a**) Nakaya, (**b**) Nakamichi, (**c**) Tenshoji and Tōkai sites (**d**) Ushimaki, (**e**) Mamizuka and (**f**) Asahi. Ranges (68% confidence) of 138 modern authentic reference products are shown, Marine, Wild boar, Freshwater, C_3_ nuts, Rice, Ruminant and C_4_ Millet. These references are from previously published data and new isotopic ranges for rice and millet obtained in this study (Online resource 3) (Craig et al. 2013; Horiuchi et al. 2015; Lucquin et al. 2016b; Heron et al. 2016; Taché et al. 2021)

To further characterise the sources of the extracted lipids, 207 samples with measurable concentrations of C_16:0_ and C_18:0_ fatty acids were analysed using GC-c-IRMS and measured against corrected values obtained from known archaeological samples and modern Japanese products (Fig. 5). The carbon isotope value (δ^13^C) of these fatty acids reflects the average contribution from different sources, weighted for their original concentrations, as such is highly sensitive to mixing and is biassed towards higher lipid content products (e.g. animal products). Samples with fatty acid isotope values entirely consistent with rice (C_3_) and millet (C_4_) were absent except for a single sample from Asahi (ASA_089), which plotted within the range of modern rice oils, but also C_3_ plants more generally. The fatty acid isotope range for ancient Japanese rice and millet was confirmed by the additional analysis of 13 modern organic rice and millet samples from Japan and the Himalayas, corrected for the burning of fossil fuels. Alongside values obtained from 2 food crust samples from the site of Joto, Japan which contained macrofossils of rice grains, yielding an average δ^13^C_16:0_ and C_18:0_ value of − 33.3‰ and − 32.1‰, respectively (Online Resource 3).

Millet appears to always be mixed with other commodities, as all samples that yielded miliacin (Nakamichi, Tenshoji and Asahi) also yielded fatty acid isotope values outside the range for authentic millets. It is difficult to understand the extent of these mixtures due to a variety of possible isotopic endpoints (freshwater, C_3_ plant, wild boar, deer). However, the isotope values obtained from Tenshoji in conjunction with the molecular data shows mixing between millet, ruminant adipose fats, and aquatic products.

The overall distribution of δ^13^C values between the pre-agricultural and post agricultural sites shows little change. However, the range of δ^13^C values in both Tōkai and the Central Highlands are distinct. δ^13^C values from pottery from Central Highland sites remain restricted in its range, likely reflecting the processing of wild terrestrial animals and freshwater resources, with no δ^13^C values indicating the processing of marine resources. Hunting of wild game by farmers in the mountainous highlands (‘inland farmers’) has been proposed by Takahashi (2009). Mixing of millet and wild ruminants such as deer, particularly in Yayoi samples from Tenshoji suggests that wild ruminants might have been the culinary staple, with the incorporation of millet into these recipes. Isotope values obtained from Nakaya and Nakamichi pottery vessels and aquatic biomarkers from Tenshoji pottery indicate that freshwater/brackish fish continued to be an important resource in the Central Highlands. Either recovered from nearby lakes and rivers or recovered from paddy fields after their introduction (Nakajima 2006).

In contrast, δ^13^C values from all sites in the Tōkai region are more broadly dispersed, reflecting a wider range of resources being utilised and processed in the ceramic containers, which we interpret as a higher input of marine resources yielding more enriched δ^13^C_16:0_ values (Fig. 5). These results agree with other lines of evidence that suggest access to marine resources would have likely continued from the Jōmon period at the coastal plains (Takahashi 2009). The continued exploitation is shown in both the biomolecular and isotopic values obtained from pottery extracts from all Tōkai sites. It is also likely that wild ruminants were exploited in both pre-agricultural and agricultural sites, where it has been proposed that in the latter farmers took advantage of wild game that entered their site as opposed to active ‘task-specific’ hunting in the Central Highlands (Takahashi 2009). Furthermore, freshwater fishing was also utilised even at locations with access to marine resources, such as at the site of Asahi where freshwater and brackish fish remains have been identified (dominated by Cyprinid spp.) (Nakajima et al. 2008). Although it is not possible here to observe an increase in the utilisation of freshwater resources after the introduction of paddy fields, again it is possible that freshwater fish were exploited through paddy fields or collected from freshwater sources during spawning season (Nakajima 2006; Nakajima et al. 2019).

### Pottery use shift at the Asahi site

**Fig. 6:**
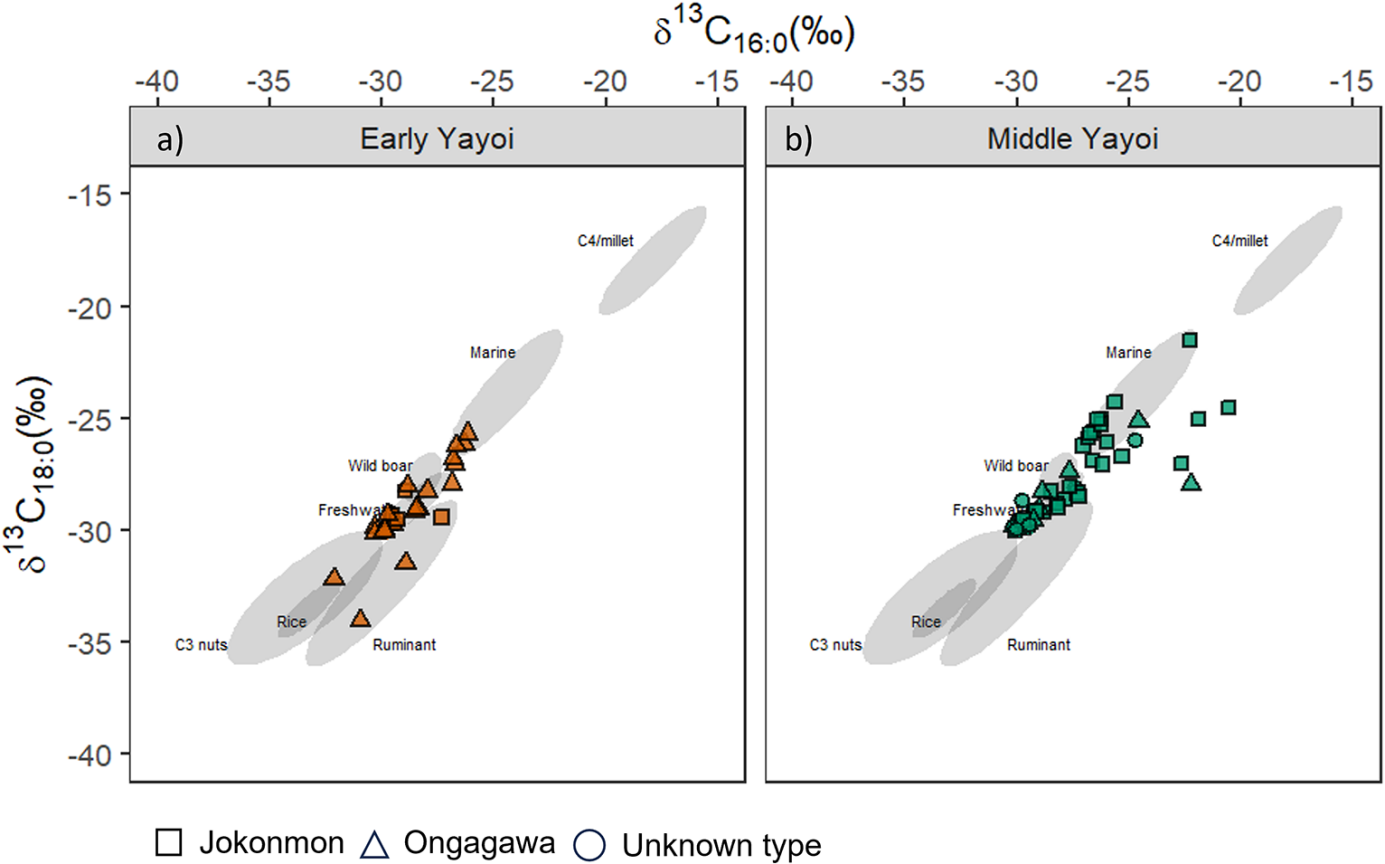
Plot of δ^13^C_16:0_ against δ^13^C_18:0_ of pottery extracts from (**a**) Early Yayoi and (**b**) Middle Yayoi pottery at the Asahi site. Ranges (68% confidence) of 138 modern authentic reference products are shown, Marine, Wild boar, Freshwater, C_3_ nuts, Rice, Ruminant and Millet. These references are from previous published data and new isotopic ranges for rice and millet obtained in this study (Online resource 3) (Craig et al. 2013; Horiuchi et al. 2015; Lucquin et al. 2016b; Heron et al. 2016; Taché et al. 2021)

As a key site, δ^13^C values from pottery from both Early and Middle Yayoi contexts at Asahi were compared to further examine the use of pottery between these two phases (Fig. 6) and between known *Jokonmon* and *Ongagawa* vessels. Here, the only evidence of C_3_ plant utilisation comes from an *Ongagawa* vessel from the early phases at Asahi (ASA_089), which could indicate the first direct evidence for the processing of rice in these vessel types. Although other C_3_ plants (e.g., acorn) cannot be entirely dismissed, a high isomeric E/H APAA-C_18_ value (> 8) further supports the processing of rice. However, δ^13^C values from the majority of *Ongagawa* types sampled from both Early and Middle Yayoi phases indicate mixtures of marine, freshwater and terrestrial animal products, not distinguishable from *Jokonmon* vessels. In comparing values from Early Yayoi to Middle Yayoi there is a significant shift in the distribution of δ^13^C values (W = 592.5, p-value = 0.0259) in the latter period. More enriched δ^13^C_16:0_ values (> -27‰) could indicate a shift to a more marine signal, which is further supported by a relatively high SRR ratio in a number of these samples (Online Resource 2). In addition, high Δ^13^C values (δ^13^C_18:0_ - δ^13^C_16:0_ ) in at least 4 samples might indicate the processing of marine resources mixed with more ^13^C depleted sources such as terrestrial animals or C_3_ plants. However, it must be noted that mixing of C_4_ plants instead of marine could also contribute to these values, but in the absence of other biomarkers (e.g., miliacin) this is not considered the most likely interpretation.

**Fig. 7:**
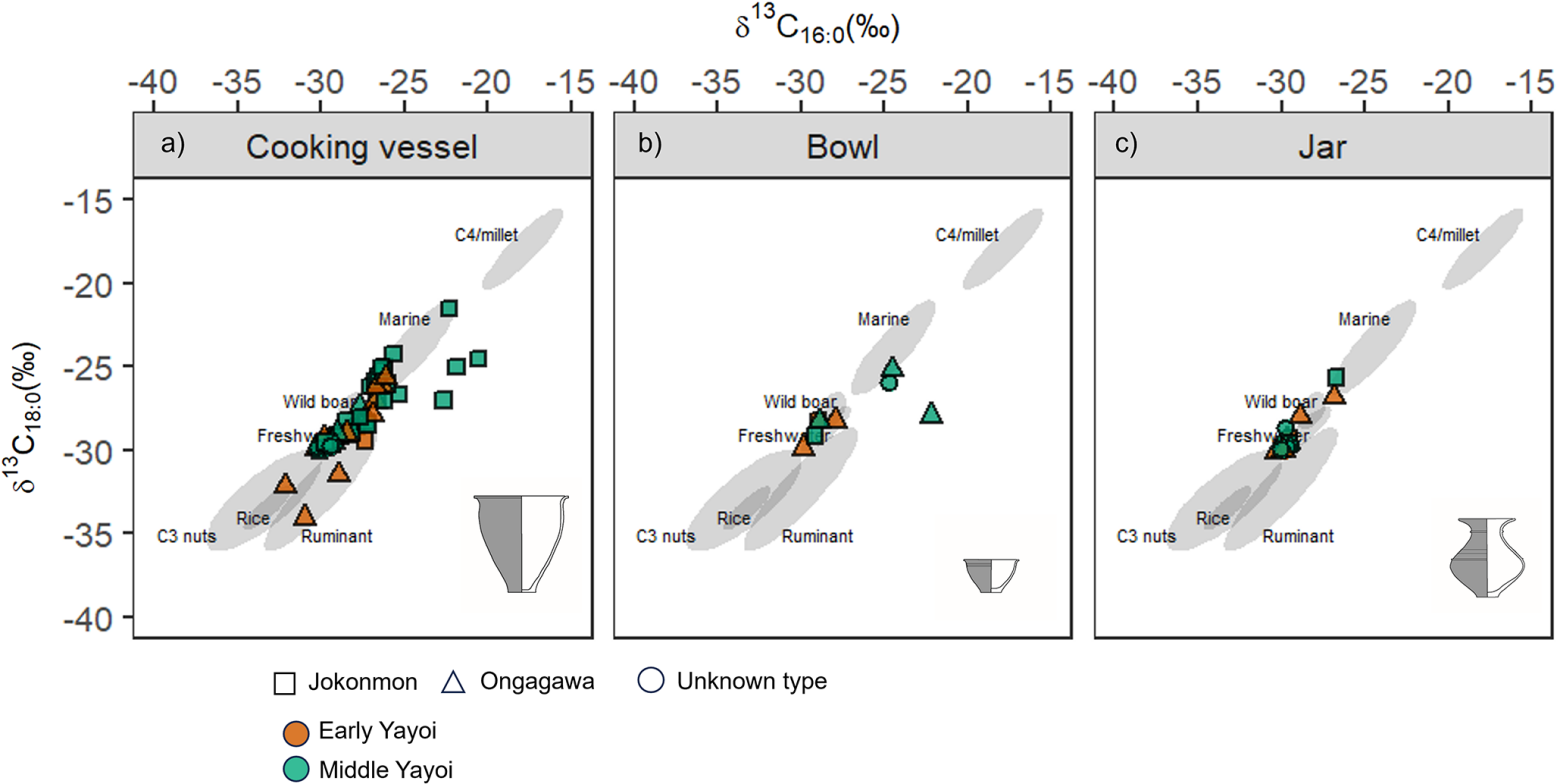
Plot of δ^13^C_16:0_ against δ^13^C_18:0_ of pottery extracts from the Asahi site (**a**) Cooking vessels, (**b**) Bowls, (**c**) Jars. Ranges (68% confidence) of 138 modern authentic reference products are shown, Marine, Wild boar, Freshwater, C_3_ nuts, Rice, Ruminant and Millet. These references are from previous published data and new isotopic ranges for rice and millet obtained in this study (Online resource 3) (Craig et al. 2013; Horiuchi et al. 2015; Lucquin et al. 2016b; Heron et al. 2016; Taché et al. 2021)

Various forms of pottery (cooking pots, bowls, and jars) of both *Ongagawa* and *Jokonmon* styles obtained from Asahi were compared to further understand if certain forms had specific uses (Fig. 7). *Tsubo* Jars represent a new vessel form that was introduced in the archipelago at the beginning of the Yayoi period (Fig. 7c). Perhaps unsurprisingly, cooking vessels showed the broadest distribution in δ^13^C values, indicating a wide range of processed products. In contrast, bowls and jars showed a more restricted range of δ^13^C values, which might suggest a dedicated use for the processing of specific resources (Kruskal-Wallis chi-squared = 8.95, df = 2, p-value = 0.01139). However, sample size and effect size (0.07) are limited, and a larger sample size would be needed to further understand the extent by which these forms differed in their use (GitHub repository).

## Assessing the arrival and impact of rice and millet agriculture in central Japan

Our results have provided direct chemical evidence for the presence of millet in late Final Jōmon pottery and Early Yayoi pottery in the Central Highlands and Early to Middle Yayoi pottery in Tōkai. For the first time, we are able to show that millet was indeed being processed for culinary purposes at these sites. Nonetheless, in line with evidence from seed impression data, the frequency of miliacin in over 250 samples analysed in this study is considerably lower than might be expected if millet was a dominant crop used for consumption. Comparable datasets are rare, so it is difficult to assess the degree to which the observed frequencies are attributable to diagenesis. However, at Majeon-ri, a Mumun (Bronze Age) agricultural site in West Korea, dating to approximately the same period, miliacin was identified at a considerably higher frequency (seven out of 15 sherds) despite similar overall lipid concentrations (Heron et al. 2016). Additionally, of the seven Majeon-ri samples that produced sufficient fatty acids for stable isotope analysis, three fall within the millet range, suggesting more intensive exploitation in Korea compared to central Japan. This inference is supported by stable isotope analysis showing extreme C_4_ diets − 11.2 ± 0.8‰, *n* = 7 during the Mumum period in Korea (Choy et al. 2021). In contrast, in the Central Highlands, where δ^13^C collagen enrichment is unlikely to be attributable to marine consumption, the human stable isotope data points to a mixed C_3_/C_4_ diet indicating millets were not a major dietary source (Yoneda et al. 2021). Interestingly, even in later historic periods, bone collagen isotope values from central Japan are never as ^13^C enriched as those from the Korean Bronze Age (Yoneda et al. 1996). These lines of evidence suggest the culinary role played by millets in the Yayoi culinary traditions differs considerably from those observed in the Mumun culture, signalling a change in their use during the dispersal process of the 1st millennium BCE.

There is even less evidence for the processing of rice in the vessels analysed here. Although our ability to identify rice is hindered by a lack of specific biomarkers, the fatty acid carbon stable isotope values are not comparable with those obtained from modern references of authentic Japanese rice (Online resource 3), or other C_3_ plants, with the exception of a single *Ongagawa* vessel from Asahi. In other studies of prehistoric East Asian pottery, such isotopic measurements alongside starch/cellulose pyrolysis products have permitted the identification of starchy plants (Shoda et al. 2018). Isotopic values obtained from food crusts adhered to Late Yayoi pottery vessels from Joto, Chugoku region, Japan, suggests that the methodology deployed is sufficient to permit rice identification, provided it is the dominant input. For the vessels analysed in this study, we therefore conclude that if present at all, rice must have been mixed with other (animal) products and that there is only very limited evidence that pottery vessels were dedicated for rice processing. In any case, our evidence supports the low frequency of rice impressions and supports the view that rice had not yet become a dominant crop at the chronological period targeted in this study.

Overall, the organic residue analysis evidence for low-level millet and rice processing and evidence for wild ruminant and aquatic resources in pottery during the early phases of agricultural adoption is most likely representative of wider subsistence practices. This indicates that communities associated with the earliest evidence of rice and millet, including Early Yayoi, were still, to some extent, following predominantly Jōmon lifeways. Fishing and hunting tools, shell middens and terrestrial faunal remains found at Yayoi sites in central Japan, alongside high proportions of wild plants (e.g., chestnuts and acorns) at sites that yield millet and rice remains, offers evidence for the continued exploitation of wild resources (Board of Education of Aichi Precture 1982; Tanaka 1988; Motoura Sites Archaeological Research Group 1995; Nakajima et al. 2008; Stevens et al. 2022). Our data not only supports this but shows that there was no radical departure in culinary traditions even after the introduction of new types of pots traditionally associated with agriculture (*Ongagawa*), breaking the common associations between pottery and economy.

We cannot dismiss the utilisation of Yayoi pottery by Jōmon hunter-gatherers. Evidence of exchange of Yayoi *Ongagawa* vessels between farmers and hunter-gatherers has been suggested in Gumma Prefecture (Barnes 2019). However, in the case of Asahi, an alternative explanation for the continued exploitation of wild resources in Yayoi cooking pots is that early farming groups acquired the indigenous knowledge from Jōmon hunter-gatherers, either with or without genetic admixture, to aid their expansion to new territories and adapt a mixed foraging, hunting, fishing and farming economy contingent on the local environmental setting. A similar explanation has been proposed for the continuation of aquatic products found in pottery produced by North European farming groups as they expanded to the Baltic coast (Lucquin et al. 2023). For the earlier agricultural sites selected in this study, we might argue that we have captured a time where reliable agricultural systems were not fully developed, and that the utilisation of local resources was particularly important at this time for mitigating the risk of failed crop yields.

This study has provided key insights into the utilisation of resources at the turn of the agricultural transition in central Japan and it should be noted that a multi-faceted approach is essential for detecting a broad range of products including rice and millet in pottery. To gain further insight into the use of rice and millet in pottery in and outside of Japan, future developments of organic residue methods should focus on the identification of starchy products in pottery vessels. Importantly, to enable a wider investigation of the impact of agriculture in the Japanese archipelago, in the next steps it is crucial to assess whether rice emerges as a dominant product in pottery in later phases or, indeed, in other regions of the archipelago to see whether a fully agrarian economy can be identified with organic residue analysis.

### Electronic supplementary material

Below is the link to the electronic supplementary material.

Online Resource 1: Materials and Methods

Online Resource 2: All samples and summary of organic residue analysis results

Online Resource 3: Modern references and experiments

## Data Availability

The main data is provided within the manuscript or supplementary information files of this article and additional data supporting the findings of this study have been deposited in the GitHub repository: 10.5281/zenodo.11164557.
