# Supplementary material for: Culinary continuity in central Japan across the transition to agriculture
Source: Archaeol Anthropol Sci. 2024 Jun 7;16(7):97. doi: 10.1007/s12520-024-01992-9 (PMC11156723; doi:10.1007/s12520-024-01992-9)
Supplement: Supplementary file 1 — Online Resource 1: Materials and Methods [file 12520_2024_1992_MOESM1_ESM.docx]

# Materials and methods

## Frequency of millet and rice impression on ceramics

Endo and Leipe [(2022)](https://paperpile.com/c/CnIiZL/STNg/?noauthor=1) have recently provided a comprehensive review study of seed impressions of rice, broomcorn millet, and foxtail millets on Late/Final Jomon to Middle Yayoi ceramics across the Japanese archipelago. Here we examine a subset of their data comprising 42 sites from the Chubu Highlands and the Tokai region (see Table 1 in the main paper for sample sizes per period and region). We considered only sites meeting the following criteria: 1) located in either Chubu or Tokai region; 2) those than can be clearly assigned to either Late Jomon to Initial Yayoi, Early Yayoi, or Middle Yayoi period; and 3) has data on the total number of sherds examined. Our final sample reflects the examination of over 35,000 potsherds, although with considerable variability in the number of samples examined in each site (ranging between 2 to 9,364 sherds).

We examined variations in the number of seeds impressions for rice and two species of millets by fitting a negative binomial model (to account for overdispersion) using as offset the number of sherds and as predictor an interaction between the nominal variables *period* (three levels: Late Jomon to Initial Yayoi, Early Yayoi, and Middle Yayoi) and *region* (two levels: Chubu Highlands and Tokai). The regression model was fitted using *brms* [*(Bürkner 2017)*](https://paperpile.com/c/CnIiZL/HjN6) using four chains and 5,000 iterations. All parameters achieved a good convergence (Rhat <1.01) and sufficient effective sample size (>2000).

## Rice agriculture arrival time

Arrival dates of rice agriculture in Tokai and Chubu Highlands were estimated by using a hierarchical Bayesian model on ^14^C dates obtained from charred rice grains. We employ the same methods and a subset of the data presented in a previous work by Crema et al [(2022)](https://paperpile.com/c/CnIiZL/bbmE/?noauthor=1) using a different geographic subdivision to specifically focus on two regions examined here. More specifically we divided the Japanese islands into the following five regions: Kyushu (1), Chugoku and Shikoku (2), Kansai (3), Tokai (4), and Chubu Highlands (5). The remaining regions of the Japanese islands were excluded from our analyses.

The Bayesian approach presented in Crema et al [(2022)](https://paperpile.com/c/CnIiZL/bbmE/?noauthor=1) estimated the occupation length of and start date for each site and subsequently models the regional arrival time using the estimated values of the latter (see Table S1 for sample sizes). The distribution of site-level start dates of each region was modelled as uniform distribution. The start dates ν of these distributions (our parameters of interest) were constrained to reflect the putative dispersal process of rice agriculture in Japan based on previous studies and the results in Crema et al [(2022)](https://paperpile.com/c/CnIiZL/bbmE/?noauthor=1). More specifically, we assumed the following three constraints:

*c1*. ν_Kyushu_ > ν_Chugoku & Shukoku_ > ν_Kansai_

*c2*. ν_Kansai_ > ν_Tokai_

*c3*. ν_Kansai_ > ν_Chubu Highlands_

We assumed a wave of advance model for the dispersal of rice farming from northern Kyushu to Kansai (*c1*) but we only assumed that the arrival time in Tokai and Chubu Highlands to be *after* Kansai (c2 and c3). In other words, we have not assumed a priori that rice arrived before in any of the two regions.

| Region | n_dates_ | n_sites_ |
| --- | --- | --- |
| Kyushu | 81 | 41 |
| Chugoku and Shikoku | 27 | 18 |
| Kansai | 26 | 6 |
| Tokai | 5 | 5 |
| Chubu Highlands | 27 | 11 |
| TOTAL | 166 | 81 |

Table S1: Number of sites and charred rice remains used for estimating rice arrival dates

Arrival times were estimated using the *NIMBLE* [(de Valpine et al. 2020)](https://paperpile.com/c/CnIiZL/EywQ) and *nimbleCarbon* [(E. R. Crema 2022)](https://paperpile.com/c/CnIiZL/kG8L) R packages using four chains, each with 6 million iterations (half discarded for burn-in) and the thinning parameter set to 300 steps. All parameters achieved a good convergence (Rhat <1.01) and the overall agreement (90.1%) suggests a consensus between the model priors and posteriors.

# Organic residue analysis methods

## Preparation and extraction

Following common protocol for the extraction of absorbed residues, each ceramic sample was first ‘cleaned’ by removing the outer layer of the ceramic surface before collecting approximately 1-2g of ceramic powder by drilling into the pot.

An acidified methanol extraction (AE) method was applied to all samples following common procedure after [(Correa-Ascencio and Evershed 2014)](https://paperpile.com/c/CnIiZL/Jj8x). A mixture of methanol (4 mL) and sulphuric acid (800 µL) was added to 1 g of ceramic powder alongside an internal standard (10 µL of C_34:0_) before undergoing 4 hours of heating at 70°C. The sample was centrifuged, and the supernatant extracted from the pottery powder and transferred to a clean labelled hatch tube. The lipid extract was separated from the acid in hexane and the supernatant passed through a filter pipette to neutralize the sample with potassium carbonate (K_2_CO_3_). The samples were dried under a gentle stream of nitrogen, and then dissolved in *n*-hexane and transferred to an auto-sampling vial with a micro insert. A second internal standard (10 µL of C_36:0_) was added before analysis by GC techniques.

A solvent extraction (SE) was undertaken on 129 samples where it was possible to retain a further 1g of powder and samples were selected to represent a variety of results obtained by AE. A mixture of dichloromethane-methanol (DCM 2 : 1, v/v) was added to 1 g of ceramic sample. The samples were sonicated for 15 minutes and then centrifuged. The supernatant was transferred to a clean labelled hatch tube. These steps were performed three times. The solution was then dried to completion under a gentle stream of nitrogen.

The dried extracts from SE and some selected AEs were derivatized by adding *N,O*-bis(trimethylsilyl)trifluoroacetamide (BSTFA) with 1% trimethyl-chlorosilane and heated for 1 hour at 70°C. *n*-hexane was added to redissolve the extract before transferring to an auto-sampling vial which contained 10 µL of the second internal standard C_36:0_ for GC analysis.

## Gas Chromatography- Flame Ionization Detector (GC-FID)

AEs and SEs were screened using a GC fitted with a flame ionization detector (FID) for quantification and general screening of preservation. An Agilent 7890A Series gas chromatograph (Agilent Technologies, Cheadle, Cheshire, UK) fitted with a DB1-high temperature (HT) column (15 m × 0.32 mm × 0.1 µm). 1 μL of the extract was injected via a splitless injector maintained at a temperature of 300°C. The temperature program was set at 100°C for 2 minutes, which rose by 20°C/min until 325°C, where it was held for 3 min. For SEs, the temperature of the column was kept at 50°C for 2 min and then increased by 10°C every minute until a final temperature of 375°C was reached. A temperature of 375°C was then held for 10 min.

## Gas Chromatography- Mass Spectrometry (GCMS)

Extracts were also analysed by GC-MS using a Shimadzu GCMS-QP2010Ultra. The inlet temperature was set to 300°C and 1 μL sample was injected using the splitless injection method. An Ultra ALLOY-5 (Frontier Laboratories Ltd., Japan: 30 m × 0.25 mm, film thickness 0.25 μm) column was used. The oven temperature was set to 50°C for 1 minute, raised to 280°C at 20°C every minute, then raised to 325°C at 5°C every minute, and held for 8.5 minutes. The ion source temperature was set to 230°C and ionization voltage to 70 eV. Helium was the carrier gas, with a flow of 3 mL per minute. A separate temperature program and selected ion monitoring (SIM) mode was used to target different ion groups of miliacin (*m/z* 189, 204, 231, 425, 440). Alternatively, GC-MS analysis was performed on a Trace Ultra gas chromatograph with a ISQ mass spectrometer (Thermo Fisher). 1 μL of sample was introduced to the GC-MS using the splitless injection method. A DB5-ms (5%-phenyl)-methylpolysiloxane column (30 m × 0.32 mm × 0.25 µm; J&W Scientific, Folsom, CA, USA) was used. The splitless injector and interface were maintained at 300°C and 340 °C respectively. Helium was the carrier gas at constant inlet pressure. The ionisation energy was 70 eV and spectra were obtained by scanning between *m/z* 50 and 800. The oven temperature programme was set to 50°C for 2 minutes and raised by 10°C every minute until a temperature of 325°C was reached. The final temperature of 325°C was then held for 15 minutes.

AEs were also analysed using an Agilent 7890A series chromatograph attached to an MS Agilent 5975 Inert XL mass selective detector with a quadrupole mass analyser (Agilent Technologies, Cheadle, UK) equipped with a DB-23 (50%-Cyanopropyl)-methylpolysiloxane column (60 m × 0.250 mm × 0.25 µm; J&W Scientific, Folsom, CA, USA). The temperature of the column was kept at 50°C for 2 minutes and then increased by 10°C every minute until 100°C. The temperature increased then until 140°C by 4°C every minute, then until 160°C by 0.5°C every minute and finally until 250°C by 20°C every minute. A SIM mode was used to target different groups of ions. These groups were: *m/z* 74, 105, 262, 290, 318 and 346 for the detection of ω-(o-alkyl phenyl)alkanoic acids of carbon lengths C_16_ to C_22_ (APAA_16–22_), *m/z* 74, 87, 213, 270 for TMTD, *m/z* 74, 88, 101, 312 for pristanic acid, *m/z* 74, 101, 171, 326 for phytanic acid.

For SEs, a HT column and programme were used to detect the presence of tri-, di and mono-acylglycerols (TAGs, DAGs and MAGs) and wax esters. A Perkin Elmer Clarus 690 GC coupled to a SQ8-T Mass Spectrometer (PerkinElmer Inc., USA) was used equipped with a DB5-HT column (30 m × 0.25 mm × 0.1 µm). 1 μL of the extract was injected directly onto the column. The temperature of the column was kept at 50°C for 2 min and then increased by 10°C every minute until a final temperature of 375°C was reached. To target ions specific to alkylresorcinols SEs were analysed using the same chromatographic conditions with the mass spectrometer in SIM mode. The ions *m/z* 73, 268, 464, 492, 520, 548, 576, 604 and 632, corresponding to alkylresorcinols with cyclic carbon chain lengths C_17_ to C_25_, were monitored.

## Gas Chromatography-combustion-Isotope Ratio Mass Spectrometry (GC-c-IRMS)

Stable carbon isotope (*δ*^13^C) values of the major saturated fatty acids (FA; C_16:0_ and C_18:0_) were analysed by GC-c-IRMS. Either a Delta V Advantage isotope ratio mass spectrometer (Thermo Fisher, Bremen, Germany) linked to a Trace Ultra gas chromatograph (Thermo Fisher) with a GC Isolink II interface was used or an Isoprime 100 (Isoprime, Cheadle, UK) with a Hewlett Packard 7890B series GC (Agilent Technologies, Santa Clara, CA, USA) and an Isoprime GC5 interface (Isoprime Cheadle, UK) was used. In both cases a a DB-5MS Ultra inert fused silica column (US, 60 m × 0.25 mm × 0.25 µm) was fitted. 1 μL of sample was injected via a splitless injector maintained at a temperature of 300°C. Helium was used as the carrier gas at a constant flow rate of 3 mL per minute. An Agilent 5975C mass spectrometer detector was attached to the column and half of the gas eluting from the column was directed to and ionized in the mass spectrometer. The other half of the gas eluting from the column was directed to the reactor tube to oxidize carbon species in CO_2_. The ionization energy of the mass spectrometer was 70 eV and ion intensities of *m/z* 44, 45 and 46 were recorded.

IonOS software was used to compute the ^13^C/^12^C ratio of the peaks in the extracts. The ^13^C/^12^C ratio was calculated in comparison with a standard reference gas (CO_2_) of known isotopic composition. The delta C values were expressed as per mil (‰) relative to the internal standard V-PDB (Vienna Pee Dee Belemnite). An *n*-alkanoic acid ester standard of known isotopic composition (F8-3) was used to determine the precision and accuracy of the instrument, which needed to remain at less than 0.5‰ and less than 0.3‰, respectively. Values were corrected in relation to the method standard, a mixture of C_16:0_ and C_18:0_ FAs of known isotopic composition.

# Statistical analysis of organic residue analysis data

Rscripts for statistical analysis of ORA data reported in the paper can be found at: <https://doi.org/10.5281/zenodo.11164557>

# References

Bürkner, Paul-Christian (2017) Brms: An R Package for Bayesian Multilevel Models Using Stan. Journal of Statistical Software [80: 1–28.](http://paperpile.com/b/CnIiZL/HjN6)

Correa-Ascencio, Marisol, and Richard P. Evershed (2014) High Throughput Screening of Organic Residues in Archaeological Potsherds Using Direct Acidified Methanol Extraction. Analytical Methods [6(5): 1330–40.](http://paperpile.com/b/CnIiZL/Jj8x)

Crema, Enrico R., Chris J. Stevens, and Shinya Shoda (2022) Bayesian Analyses of Direct Radiocarbon Dates Reveal Geographic Variations in the Rate of Rice Farming Dispersal in Prehistoric Japan. [Science Advances 8(38): eadc9171.](http://paperpile.com/b/CnIiZL/bbmE)

Crema, E. R (2022) nimbleCarbon: Models and Utility Functions for Bayesian Analyses of Radiocarbon Dates with NIMBLE (v.0.2.0). <https://github.com/ercrema/nimbleCarbon.>

Endo, Eiko, and Christian Leipe (2022) The Onset, Dispersal and Crop Preferences of Early Agriculture in the Japanese Archipelago as Derived from Seed Impressions in Pottery. Quaternary International: The Journal of the International Union for Quaternary Research [623: 35–49.](http://paperpile.com/b/CnIiZL/STNg)

Valpine, P. de, C. Adler, D. Turek, N. Michaud et al (2020) [*NIMBLE: MCMC, Particle Filtering, and Programmable Hierarchical Modeling*](http://paperpile.com/b/CnIiZL/EywQ) (version 0.10.1). https://doi.org/[10.5281/zenodo.1211190](http://dx.doi.org/10.5281/zenodo.1211190)[.](http://paperpile.com/b/CnIiZL/EywQ)
